# Effects of a Group-Based Cognitive Behavioral Therapy for Sleep in Nursing Students: Pilot Randomized Controlled Trial

**DOI:** 10.2196/85021

**Published:** 2026-09-16

**Authors:** David Pérez-Manchón, Clara Azpeleta, Beatriz Gal-Iglesias, Cayetana Ruiz Zaldibar

**Affiliations:** 1 Department of Nurisng, University Center for Health Sciences – HM Hospitals (CUHMED), Camilo Jose Cela University, Villanueva de la Cañada 28692, Madrid, 01004, Spain, 34 695829472; 2 Instituto de Investigación Sanitaria HM Hospitales, Madrid, Spain; 3 Universidad Europea de Madrid, Faculty of Medicine, Health and Sports, Department of Medicine, Madrid, Spain; 4 Departamento de Ciencias Médicas Básicas, Facultad de Medicina, Universidad CEU San Pablo, CEU Universities, Madrid, Spain

**Keywords:** nursing students, sleep quality, sleep hygiene, wearable, cognitive behavioral therapy

## Abstract

**Background:**

Sleep quality is a strategic public health priority and a key factor in the study of circadian rhythms and chronotypes. Nursing students are a particularly vulnerable population group.

**Objective:**

The objective of this study was to evaluate the effects of a brief group-based cognitive behavioral therapy for insomnia (CBT-I) intervention on sleep quality and circadian-related outcomes among first-year nursing students using ambulatory circadian monitoring.

**Methods:**

This study is a 2-arm pilot randomized clinical trial conducted at a private university in Madrid, Spain, from October 2022 to March 2023. First-year nursing students aged 18 to 25 years were recruited in October 2022 using convenience sampling and randomly assigned to an experimental group (n=20) that received a cognitive-behavioral intervention using an active-constructive learning methodology to improve sleep quality and a control group (n=20) that followed their usual daily routine. Primary outcomes were objective sleep quality assessed using the Kronowise 3.0 ambulatory circadian monitoring system and subjective sleep quality assessed using the Pittsburgh Sleep Quality Index (PSQI). The objective Kronowise assessment was characterized by the continuous circadian rhythm and sleep parameters generated by the device. Participant satisfaction with the intervention was evaluated using an adapted 12-item Likert-type satisfaction questionnaire administered after the intervention. Measurements were collected preintervention and postintervention over 7-day monitoring periods. Adjusted analyses were performed using analysis of covariance, with treatment group as the fixed factor and baseline values, smoking status, school schedule, and coffee consumption as covariates. Treatment effects were summarized using regression coefficients (β), 95% CIs, and partial eta-squared (η²). The study was conducted in accordance with the CONSORT (Consolidated Standards of Reporting Trials) statement.

**Results:**

Forty students (n=34, 85% female; mean age 19.9, SD 1.8 y) were randomized equally into the intervention and control groups. Adjusted analyses showed generally small treatment effects for objective circadian rhythm and sleep outcomes. The largest treatment effect was observed for self-reported sleep latency (β=−19.78 min, 95% CI −33.83 to −5.74; partial η²=0.194). Sleep duration (β=36.32 min, 95% CI −5.01 to 77.64; partial η²=0.086) and sleep efficiency (β=8.75%, 95% CI −0.97 to 18.47; partial η²=0.090) also favored the intervention group, although CIs indicated considerable uncertainty around these estimates. Participants reported high satisfaction with the intervention, with 65% (13/20) rating the program as “very good.”

**Conclusions:**

This pilot randomized controlled trial provides preliminary evidence that a brief group-based CBT-I intervention may improve selected subjective sleep outcomes, particularly sleep latency, among first-year nursing students. Objective circadian rhythm and sleep outcomes showed generally small treatment effects. Given the exploratory nature of this pilot study, adequately powered randomized controlled trials with longer follow-up are needed to confirm these preliminary findings.

## Introduction

Sleep is an essential physiological process for physical restoration and cognitive functioning. Sleep quality is closely linked to circadian rhythm regulation and is a key determinant of physical and psychological health. Adequate sleep quality is associated with well-being and daytime functioning, whereas poor sleep quality has been linked to adverse health outcomes, including anxiety, depression, hypertension, and metabolic disorders [1-3]. Because sleep quality reflects both behavioral and circadian processes, it is an important indicator for understanding sleep-wake regulation and overall circadian health [4].

It is estimated that 1 in 3 adults has a sleep disorder [5], and university students are a particularly vulnerable population due to academic demands, irregular schedules, and lifestyle-related factors that may disrupt healthy sleep patterns. Poor sleep quality in this population has been associated with fatigue, impaired concentration, emotional distress, and poorer academic performance [6]. Nursing students may be especially vulnerable because of the demanding academic workload, early clinical exposure, and the need to adapt to unfamiliar practice environments, all of which may increase stress and contribute to sleep disruption [7,8]. This vulnerability may be particularly relevant during the first academic year, when students are adapting to university life while facing increased academic and professional expectations.

Given the limitations of pharmacological approaches for sleep problems, nonpharmacological strategies have gained increasing attention [9]. Cognitive behavioral therapy (CBT) is supported by strong evidence as an effective intervention for improving sleep outcomes [10], and psychoeducational approaches have shown promise in promoting healthy sleep behaviors in nonclinical populations [11,12]. In university settings, psychological interventions have often focused on sleep-related symptoms associated with anxiety or depression rather than on sleep health promotion itself [13]. In addition, most studies have relied primarily on self-reported outcomes, with limited attention to objective indicators of circadian functioning such as motor activity, thermoregulation, and environmental synchronization (ES) [14]. This gap is particularly relevant for nursing students, who frequently experience sleep disturbances [15] and may benefit from behavioral interventions targeting maladaptive sleep-related habits and beliefs [16]. Although the efficacy of cognitive behavioral therapy for insomnia (CBT-I) is well established in clinical populations, less is known about its effects among nonclinical university students when delivered in group-based formats. In addition, most intervention studies in this field rely primarily on measures such as the Pittsburgh Sleep Quality Index (PSQI) [17]. Ambulatory circadian monitoring (ACM) offers the possibility of continuously assessing sleep-wake patterns and circadian regulation in real-world settings, providing a more comprehensive evaluation of behavioral intervention effects than self-report measures alone [18-21].

Although there are studies associated with poor sleep quality in nursing students [15], the evidence regarding the feasibility and acceptability of brief, group-based CBT-I interventions implemented as part of university health promotion programs for first-year nursing students remains limited. Besides, no previous studies have designed and evaluated interventions to improve sleep quality in this group using a wearable wristband device. Therefore, this pilot study aimed to explore the preliminary impact of a group-based CBT intervention on sleep quality among first-year nursing students. Specifically, the objectives were (1) to explore the preliminary effects of the intervention on sleep quality outcomes and (2) to assess participant satisfaction with the intervention. We hypothesized that, compared with the control group, participants receiving the intervention would show preliminary improvements in sleep quality from baseline to postintervention, as assessed through ACM and self-reported sleep quality.

## Methods

### Design

This study is a single-center, single-blind (data analysts), 2-arm pilot randomized clinical trial comparing 2 conditions: the intervention group, which receives a CBT-based intervention targeting sleep quality, and the control group, which receives no intervention. The randomization procedure was performed using a computer-generated random number sequence (Sealed Envelope software, version 1.21.0) and was assigned to participants’ academic record numbers to randomly designate participants to 1 of the 2 groups (intervention or control group). Random permuted blocks were also used to reduce the predictability of the random sequence and guarantee a 1:1 allocation ratio. This sequence was stored in a password-protected table and was hidden from other researchers during the study.

The study is registered at ClinicalTrials.gov with reference number NCT05273086, and the study protocol has been published [22]. This study was performed according to the CONSORT (Consolidated Standards of Reporting Trials) statement (Checklist 1).

### Study Setting, Recruitment, and Sampling

The study was conducted at a private university located in Madrid (Spain) during the 2022 to 2023 academic year. Participants were recruited in October 2022, and the study lasted from October 2022 to March 2023, when the postmeasure tests were carried out.

A convenience sampling method was used. Eligible students were informed about the study through an in-class presentation delivered by the research team, during which the study aims, procedures, duration, eligibility criteria, voluntary nature of participation, and confidentiality safeguards were explained. Students who expressed interest in participating were screened according to the inclusion and exclusion criteria. Those who met the eligibility criteria and agreed to participate provided written informed consent before baseline assessment and randomization.

The eligibility criteria of this study were as follows: the inclusion criteria were students aged between 18 and 25 years who were enrolled in the first year of a nursing degree during the 2022 to 2023 academic year. Participants were excluded if they were diagnosed with a prior mental pathology, a sleep disorder, or both, and were treated with or without medication (hypnotics, sedatives, or melatonin); were taking medications to aid sleep; or were combining work and study.

Participants were not restricted to individuals with clinically diagnosed sleep disorders because the intervention was conceived as a universal sleep health promotion program for first-year nursing students. The objective was to promote healthier sleep-related habits and behaviors in a population exposed to academic demands and future shift-work schedules, rather than to diagnose or treat clinical or subclinical sleep disorders. This approach is consistent with current strategies aimed at promoting healthy sleep at the population level [23].

### Sample Size Calculation

This study was designed as a pilot randomized controlled trial. Pilot studies are intended to assess feasibility and estimate key parameters for the design of a definitive trial, such as outcome variability and effect size estimates. Therefore, a formal a priori sample size calculation based on statistical power was not performed.

The methodological literature [24,25] on pilot studies suggests sample sizes ranging from 12 to 35 participants per group for this type of design. Although the protocol planned to recruit 60 participants, only 40 eligible students were enrolled during the recruitment period. This sample size also allows for the estimation of preliminary parameters with reasonable precision, in line with approaches focused on estimation rather than hypothesis testing. The findings of this study may inform the sample size calculation of future fully powered randomized controlled trials.

### Intervention

#### Control Group

The control group continued with its normal routine. They did not attend any sessions; they only completed the pre-post measurements.

#### Intervention Group

Participants allocated to the intervention group received a structured group-based CBT-I–informed program specifically adapted for first-year nursing students within a university health promotion context. The intervention protocol was developed by the research team based on established cognitive behavioral sleep intervention principles and was previously described in the published study protocol [22]. The protocol integrated evidence-based components, including sleep psychoeducation, cognitive restructuring, behavioral stimulus control, and sleep hygiene strategies, delivered using active-constructive learning methodologies designed to promote participant engagement and self-reflection. The intervention group was subdivided into 2 groups of 10 participants to facilitate interaction and individualized discussion. The program consisted of 2 face-to-face 90-minute sessions delivered over 2 consecutive weeks during November and December 2022.

The first session focused on sleep psychoeducation and cognitive restructuring, including basic concepts of sleep physiology and circadian regulation, identification of biological and behavioral factors influencing sleep, and guided self-assessment of individual sleep patterns and habits. Participants were encouraged to reflect on their own sleep-related behaviors between sessions. The second session focused on behavioral skill development, including sleep hygiene education, stimulus control strategies, and practical behavioral adaptations tailored to participants’ self-reported routines and baseline sleep assessments.

The intervention was delivered by a PhD candidate in biology, specialized in sleep neurophysiology, chronobiology, and circadian rhythms, and by a registered nurse (PhD) with expertise in health promotion and healthy lifestyle education. Both providers had training in sleep health and cognitive behavioral strategies for sleep. Intervention materials and procedures were reviewed by the research team before implementation to ensure consistency with the published protocol. Additional details regarding session content, intervention materials, and implementation procedures are provided in Multimedia Appendix 1.

### Outcome Measures

#### Primary Outcomes

In accordance with the trial registration (ClinicalTrials.gov NCT05273086), the prespecified primary outcomes were objective sleep quality, assessed using the Kronowise 3.0 ACM system, and subjective sleep quality, assessed using the PSQI.

The Kronowise system provides a multidimensional assessment of sleep quality and circadian function through validated continuous circadian rhythm and sleep parameters, rather than a single global categorical score. Accordingly, the objective sleep outcome was characterized using these continuous parameters, which together describe sleep quality and circadian health.

In this study, sleep and circadian rhythms were monitored using the Kronowise 3.0 device (Kronohealth, S.L.) for 7 consecutive days (including weekends) in participants’ homes during the preintervention period and for another 7 consecutive days during the postintervention period, in both the intervention and control groups, with a difference of 19 days between the 2 monitoring periods. The technical characteristics of the device have been described in several studies [14,26,27], and the analysis of its monitoring parameters was obtained from a population of 244 healthy volunteers aged 14 to 86 years using the Kronowizard platform and the Circadianware program, following the methodology of Madrid-Navarro et al [27] from the University of Murcia.

The primary signals recorded by the Kronowise system were surface temperature, acceleration, position, and exposure to light (TAPL). Peripheral skin temperature of the wrist is a recognized, validated circadian rhythm marker [28], suggesting that an increase in peripheral skin temperature is associated with drowsiness [29], whereas a decrease in core temperature is due to redistribution of heat from the interior to the periphery [30]. The triaxial motor acceleration shows the intensity of physical activity and the time in movement, calculated as the sum of the absolute values of 300 acceleration vectors. Changes in posture reflect postural changes according to inclination axes (horizontality vs verticality) to identify immobility and the amplitude of movements during periods of activity vs rest. Light exposure is measured in 3 spectral bands (visible, blue [460‐480 nm], and infrared [>700 nm]). All these variables were recorded at a sampling rate of 10 Hz (for acceleration and time in motion), 1 Hz (for skin temperature and light exposure), and 0.033 Hz for wrist position, generating 23 million raw data points over 7 days, condensed to 230,000, which were stored in 30-second stretches for later analysis. From these primary variables, the integrated variable TAPL is obtained as a modification of thermometry, activity, and body position, which expresses the level of general physical and mental activation and has been proved to be accurate for the estimation of individual sleep variables [20]. In TAPL, temperature is inverse to acceleration, position, and light exposure and, in global terms, acquires values between 0 and 1. Absolute values of 0 or close to 0 indicate low general activation (with high values of skin surface temperature), whereas high values close to or equal to 1 reflect high levels of activation corresponding to a maximum state of wakefulness, exposure to light, and movement (with low values of temperature). Given the objective of the investigation, TAPL V5 was used to obtain the mean value of this variable during the 5 consecutive hours of minimum values, that is, during sleep. TAPL V5 values range from 0.01 (5th percentile) to 0.07 (95th percentile), with a positive effect if it reaches lower values.

#### Circadian Parameters

The analysis of circadian parameters is related to sleep quality, and alterations may indicate chronodisruption [7]. All of these parameters range from 0 to 1, and their interpretation is based on previously published standardization procedures and normative values, including the 5th and 95th percentile reference values [20,21,26,27]. The interdaily stability (IS) regularity index, which is an indicator of the interdaily variability of the circadian rhythm, was calculated. The IS requires repeated measurements over several days because low values or values close to 0 indicate greater sleep fragmentation, whereas high values or values close to 1 indicate perfect stability. Relative amplitude normalized (RAN) is the normalized relative amplitude of the rhythm, calculated as the difference between M10 (average of the 10 consecutive hours with the maximum values of variables that rise during wakefulness) and L5 (average of the 5 consecutive hours with the minimum values of variables that fall during sleep), meaning that the higher the amplitude, the more robust the rhythm. The ES is the parameter related to the person’s chronotype and indicates the degree of synchronization between the period of sleep or rest and the center of natural darkness, taking the official summertime of the country where the participant lives as a reference. In Spain, this central time of darkness during daylight saving time is 2 AM. Low values or values close to 0 indicate that the central hour of sleep is far from the central hour of darkness, whereas high values or values close to 1 indicate that the center of sleep is close to or coincides with the central hour of darkness. The main circadian rhythm robustness parameter is the circadian health score (CHS), described in 2019 [26], which integrates IS, RAN, and ES. CHS is the primary sleep quality indicator of the ACM technique and the main marker of circadian strength, which behaves inversely to chronodisruption when it has a value close to 1, implying a high-amplitude, nonfragmented, and stable rhythm.

#### Sleep Quality Parameters

Although sleep-wake parameters were detected with the above indices, specific sleep indicators were analyzed. These indicators were temperature 2 hours before sleep, temperature during sleep, exposure to total light and blue light 2 hours before sleep, sleep latency and actual sleep time, sleep activity time, number of awakenings, and sleep efficiency. Sleep latency refers to the time in minutes to sleep onset, actual sleep time is the time spent objectively asleep, and sleep activity time is expressed as the average time in seconds during which movement is detected. The number of awakenings is the average number of movements detected for ≥30 seconds per hour of the recorded sleep interval, and sleep efficiency refers to the percentage of time asleep as opposed to the time in bed.

#### Chronotype

Chronotype refers to categories defined by the differences in activity and alertness between morning and evening. These categories are determined in relation to the 2019 Munich test, which classifies them into extreme matutine, matutine, indefinite, vespertine, and extreme vespertine [31].

#### Subjective Sleep Quality

The PSQI [17] contains 19 items and 7 clinically important components related to sleep quality: subjective sleep quality, sleep latency, sleep duration, sleep efficiency, sleep disturbances, use of sleeping medication, and daytime dysfunction. Responses are reported based on a 5-point Likert-type scale ranging from 0 to 4. Overall, sleep scores of 5 or less are considered good quality, whereas scores of 5 or more are considered low quality. The PSQI version used is validated in the Spanish university framework by de la Vega et al [32] with a Cronbach α of 0.72. The questionnaire was delivered to all participants twice (before and immediately following the intervention).

### Independent Variables

Independent variables were selected to characterize factors known to influence sleep quality and circadian regulation in young adults and university populations, including sociodemographic characteristics, lifestyle habits, physical activity, and contextual factors that may act as potential confounders or moderators of sleep-related outcomes. Satisfaction was additionally assessed to explore the acceptability of the intervention in this pilot study. Questionnaires on sociodemographic data and lifestyle factors were prepared ad hoc by the researchers.

First, sociodemographic data and anthropometric variables included date of birth, age, sex, and sleep habits during the study, including whether the participant slept alone or with someone. Participants reported their weight (kg) and height (cm) for BMI calculation. Second, lifestyle factors included tobacco consumption (cigarettes per day), alcohol consumption (daily, weekly, and sporadic), drug consumption (yes/no and type), consumption of stimulating drinks (eg, coffee, sugary drinks, and energy drinks), and consumption of nonstimulating sweetened beverages. Third, physical activity was assessed using the simplified version of the International Physical Activity Questionnaire (IPAQ) [33]. The questionnaire consists of 7 questions assessing frequency, duration, and intensity of moderate and intense physical activity during the previous 7 days, as well as walking and sitting time during a working day. The questionnaire is classified into low, moderate, and high physical activity based on metabolic equivalent task minutes per week. The higher the score, the more physically active the profile. The questionnaire has been validated, with a mean reliability of 0.80 [33]. Fourth, satisfaction with the program was assessed using a questionnaire adapted from the proposal by Azpeleta et al [34], consisting of 12 questions with responses based on a 5-point Likert scale. Higher scores indicated greater satisfaction.

### Data Collection

Data collection was conducted at 2 time points: baseline (preintervention) and immediately after completion of the intervention (postintervention). At baseline, eligible participants completed self-administered questionnaires assessing sociodemographic characteristics, lifestyle factors, physical activity, and sleep quality through the PSQI. Following questionnaire completion, participants were fitted with the Kronowise 3.0 wearable device and received standardized instructions for its use during the 7-day ambulatory monitoring period.

Participants wore the device continuously for 7 consecutive days, including weekdays and weekends, during their usual daily routines in their home environment. At the end of each monitoring period, devices were returned to the research team for data download and processing using the Kronowizard platform.

After baseline data collection, participants were randomized to either the intervention or control group. Following completion of the intervention period, both groups underwent a second 7-day monitoring period under the same conditions and completed the postintervention PSQI. Participants allocated to the intervention group additionally completed the satisfaction questionnaire immediately after the intervention. All assessments and device management procedures were coordinated by an independent investigator trained in study procedures and not involved in outcome analysis.

### Ethical Considerations

This study was approved by the Research Ethics Committee of the Camilo José Cela University (code: 06‐22-UCJC-Sleep) on April 1, 2022. All participants were informed of the duration and characteristics of the study, as well as its voluntary nature. No financial or nonfinancial compensation was provided to participants for study participation.

Participants were free to leave the study at any time, without further consequences. The utmost professionalism and absolute confidentiality were always ensured, in accordance with the European Regulation (EU) 2016/679 on the protection of natural persons and the processing and free movement of data, the Framework Law 3/2018 on the protection of personal data and guarantee of digital rights, and the Law 14/2007 on biomedical research. Only the main project researcher had access to the encrypted database, data processing, and free movement, in accordance with the Framework Law 3/2018 on the protection of personal data and guarantee of digital rights and Law 14/2007 on biomedical research. Only the main project researcher has access to the encrypted database.

### Data Analysis

A descriptive analysis was made of all the variables included in the study. Categorical variables were analyzed using absolute and relative frequencies, whereas continuous variables were described using mean (SD) if the variable followed a normal distribution or median (IQR) if the variable followed a nonnormal distribution. Normality was checked using graphic methods (histogram) and statistical methods (Kolmogorov-Smirnov test or Shapiro-Wilk test).

A bivariate analysis was performed to compare the control group and the intervention group, using the Student *t* test (1-tailed) or the Mann-Whitney *U* test for continuous variables according to the distribution of the variable and the chi-square test or Fisher exact test for categorical variables when necessary. For pre-post comparison, the Student *t* test was used for paired samples, the Wilcoxon test was used for continuous variables, and the McNemar test was used for categorical variables. Effect size for parametric comparisons was estimated using Cohen *d* [35]. For nonparametric analyses, effect size was calculated as *r* based on the standardized *Z* statistic (*r* = *Z* / √N) proposed by Rosenthal and Rubin [36], where N represents the total sample size. Effect sizes were interpreted according to Cohen conventional thresholds for *d* (0‐0.3 for small, >0.3‐0.8 for moderate, and >0.8 for large effect) and *r* (0‐0.3 for small, >0.3‐0.5 for moderate, and >0.5 for large effect).

To account for baseline imbalances between groups, adjusted analyses were additionally performed using analysis of covariance. For each continuous sleep and circadian outcome, the postintervention value was entered as the dependent variable, treatment group (intervention vs control) as the fixed factor, and the corresponding baseline value of the outcome as a covariate. Smoking status, school schedule, and coffee consumption were additionally included as covariates because these variables differed between groups at baseline. Adjusted treatment effects (regression coefficients [β]), 95% CI, *P* values, and partial eta-squared (η²) were estimated. Because baseline differences were identified between the intervention and control groups, the adjusted analysis of covariance models were considered the primary analytical approach for estimating treatment effects, whereas the unadjusted bivariate analyses were retained as complementary analyses.

In accordance with the trial registration, the prespecified primary outcomes were objective sleep quality, assessed using the Kronowise system, and subjective sleep quality, assessed using the PSQI. The continuous circadian rhythm and sleep parameters generated by the Kronowise system were analyzed to provide a detailed characterization of the objective sleep outcome. Given the pilot nature of this trial, analyses of these individual parameters were considered exploratory and hypothesis-generating. Therefore, no formal adjustment for multiple comparisons was applied, and findings from these analyses should be interpreted as exploratory rather than confirmatory.

## Results

**Figure 1.**
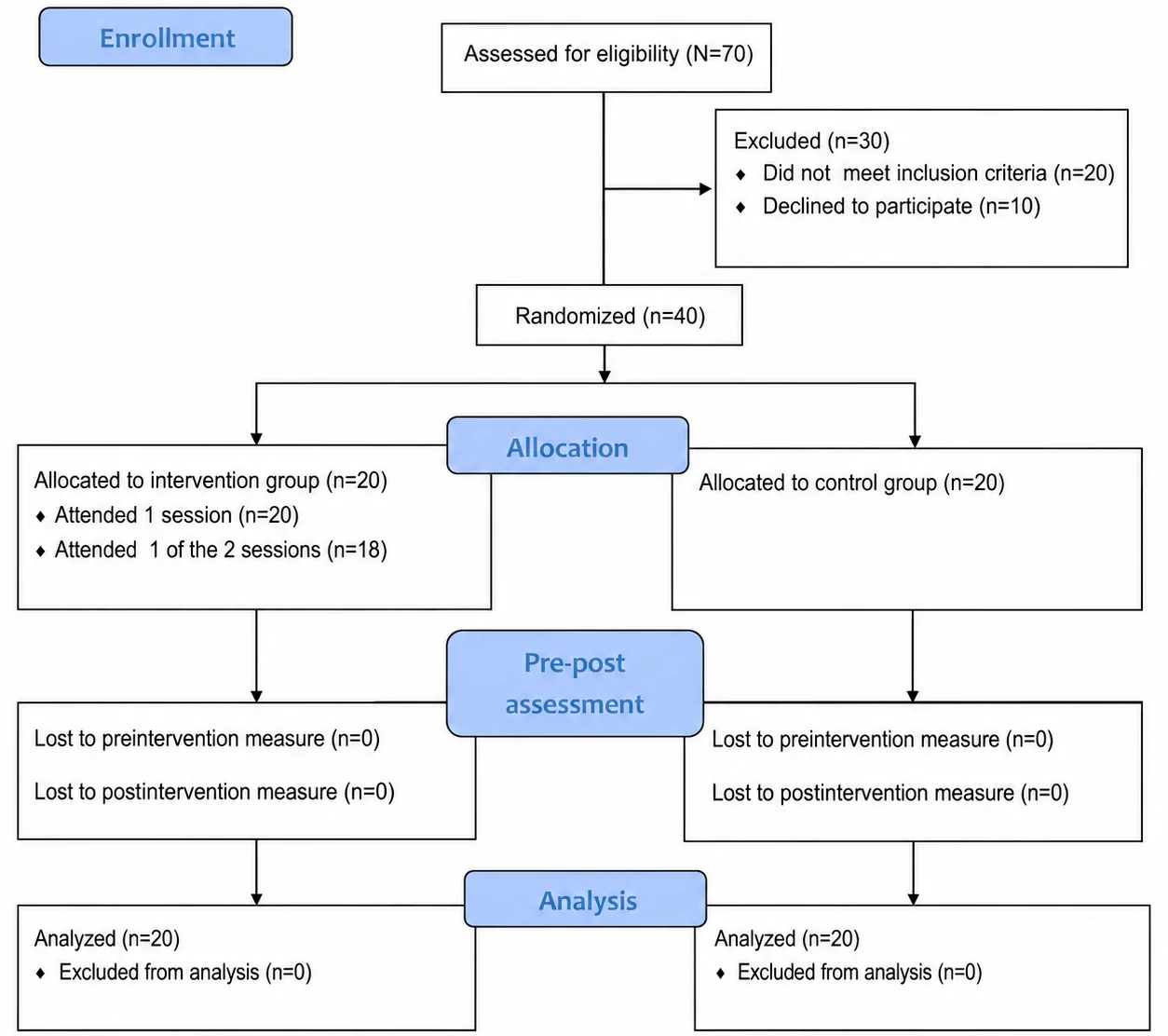
CONSORT (Consolidated Standards of Reporting Trials) diagram.

Figure 1 shows the CONSORT flow diagram of the study. A total of 70 students were assessed for eligibility. Twenty students were excluded because they did not meet the study criteria, most frequently due to melatonin use, combining work and study, or being outside the eligible age range. Of the 50 eligible students, 10 declined participation or did not complete the informed consent process. Reasons for nonparticipation were not systematically recorded. Consequently, 40 participants were enrolled and randomized. Of these, 20 were allocated to the intervention group (n=16, 80% female) and 20 to the control group (n=18, 90% female). The overall mean age was 19.9 (SD 1.8) years. The mean age was lower in the intervention group compared with the control group (mean 19.6, SD 1.8 vs mean 20.2, SD 1.8, respectively). Cohabitation of students during the study was mostly in a family setting (n=12, 60%) compared with 8 (40%) students residing outside the family environment, with no statistically significant difference.

The total sample was distributed in academic dedication schedules of 50% (20/40) morning shifts (8:30 AM to 1:30 PM) and 50% (20/40) in afternoon shifts (3:30 PM to 8:30 PM), with a statistically significant relationship (*P*=.01). The most prevalent BMI category in both groups was normal weight (31/40, 77.5%), with a mean of 23.1 (SD 3.8) kg/m^2^ (Table 1).

Among 40 students, 37.5% (n=15) were active smokers, with a higher proportion of smokers in the intervention group than in the control group, a mostly daily consumption pattern (n=10, 66.7%), and a statistically significant median daily consumption (*P*=.04). A total of 67.5% (n=27) consumed alcohol on a regular basis, with no significant differences between the 2 research groups (Table 2). Almost 8 out of 10 participants reported daily coffee consumption, with higher consumption in the control group (18/20, 90%) than in the intervention group (12/20, 60%), which was statistically significant (*P*=.03), with a median consumption of close to 2 coffees per day in both groups. Almost 3 out of 10 students reported consuming energy drinks daily, with slightly higher consumption in the control group than in the intervention group (5/20, 25% vs 4/20, 20%). Overall, 45% of the participants were sedentary, with a median of 7 hours sitting per day. The sedentary condition was the highest proportion in both groups in terms of the type of physical activity they engaged in daily.

**Table 1.** Sociodemographic data and anthropometric variables.

Sociodemographic characteristics	Global (n=40)	Intervention group (n=20)	Control group (n=20)	*P* value
Age, mean (SD)	19.9 (1.8)	19.6 (1.8)	20.2 (1.8)	.29
Sex, n (%)				.66
Female	34 (85)	16 (80)	18 (90)	
Male	6 (15)	4 (20)	2 (10)	
Class schedule, n (%)				.01
Morning	20 (50)	14 (70)	6 (30)	
Afternoon	20 (50)	6 (30)	14 (70)	
Cohabitation during the study, n (%)				.23
Alone	12 (30)	5 (25)	7 (35)	
In a relationship	1 (2.5)	0 (0)	1 (5)	
With family	24 (60)	12 (60)	12 (60)	
Shared accommodation	3 (7.5)	3 (15)	0 (0)	
BMI (kg/m^2^), mean (SD)	23.1 (3.8)	23.5 (4.3)	22.7 (3.2)	.52
BMI (kg/m^2^), n (%)				.67
Low	1 (2.5)	0 (0)	1 (5)	
Normal	31 (77.5)	16 (80)	15 (75)	
Overweight	5 (12.5)	2 (10)	3 (15)	
Obese	3 (7.5)	2 (10)	1 (5)	

**Table 2.** Baseline sociodemographic characteristics and lifestyle factors of first-year nursing students participating in a pilot randomized controlled trial evaluating a group-based cognitive behavioral therapy for insomnia (CBT-I) intervention (Spain, 2022‐2023)^a^.

Lifestyle	Global (n=40)	Intervention group (n=20)	Control group (n=20)	*P* value
With a diagnosed illness, n (%)	6 (15)	2 (10)	4 (20)	.66
On pharmacological medication, n (%)	4 (10)	1 (5)	3 (15)	.61
Smokers, n (%)	15 (37.5)	8 (40)	7 (35)	.74
Consumption (n=15), n (%)				.63
Daily	10 (66.7)	5 (62.5)	5 (71.4)	
Weekly	4 (26.7)	2 (25)	2 (28.6)	
Monthly	1 (6.7)	1 (12.5)	0 (0)	
Tobacco (n=15), n (%)				.96
Factory made	7 (46.7)	4 (50)	3 (42.9)	
Rolled cigarettes	6 (40)	3 (37.5)	3 (42.9)	
Electronic devices	2 (13.3)	1 (12.5)	1 (14.3)	
Cigarettes per day, median (IQR); range	2 (0-10); 0-10	0 (0-3.5); 0-5	10 (0-10); 0-10	.04
Alcohol consumption, n (%)	27 (67.5)	13 (65)	14 (70)	.74
Alcohol type^b^ (n=27), n (%)				
Beer	21 (77.8)	11 (84.6)	10 (71.4)	.68
Wine	15 (55.6)	8 (61.5)	7 (50)	.55
Spirits	13 (48.1)	7 (53.8)	6 (42.9)	.57
Liqueurs	4 (14.8)	3 (23.1)	1 (7.1)	.33
Alcohol volume (cm^3^/mL) per week, median (IQR); range	300 (175-600); 50‐2000	350 (212.5-800); 125‐2000	275 (125-531.25); 50‐1025	.31
Coffee consumption, n (%)	30 (75)	12 (60)	18 (90)	.03
Coffee consumption per day, median (IQR); range	0.5 (1-2); 1-4	1.5 (1-2.75); 1-4	1.5 (1-2); 1-3	.78
Daily energy drink consumption, n (%)	9 (22.5)	4 (20)	5 (25)	>.99
Energy drink types (n=9), n (%)				.46
Monster	6 (66.7)	2 (50)	4 (80)	
Caffeine	2 (22.2)	1 (25)	1 (20)	
Red Bull	1 (11.1)	1 (25)	0 (0)	
Daily consumption of non–energy-sweetened beverages, n (%)	12 (30)	5 (25)	7 (35)	.49
Beverages drink types (n=12), n (%)				>.99
Cola soft drinks	8 (66.7)	3 (60)	5 (71.4)	
Other soft drinks	4 (33.3)	2 (40)	2 (28.6)	
Hours seated per day, median (IQR); range	7 (5-9); 1-15	7.5 (5-8); 1-15	7 (4-10); (1-15)	.66
Physical activity type, n (%)				.57
Low or sedentary	18 (45)	8 (40)	10 (50)	
High	11 (27.5)	7 (35)	4 (20)	
Moderate	11 (27.5)	5 (25)	6 (30)	

a The total sample included 40 participants (20 per group). For behavior-specific variables, such as alcohol and tobacco consumption, data refer only to participants reporting the corresponding behavior; therefore, frequencies may not add up to the total sample. Continuous variables with nonnormal distributions are presented as median (Q1–Q3); range.

b For alcohol type, participants could report more than one category; therefore, frequencies and percentages do not necessarily add up to the total sample or 100%.

Adjusted treatment effects for circadian rhythm, objective sleep, and subjective sleep outcomes are presented in Table 3. For objective circadian rhythm parameters, adjusted treatment effects were uniformly small (partial η²=0.004‐0.047), and the corresponding 95% CIs were compatible with little or no intervention effect. Similarly, objective sleep and environmental outcomes showed generally small effect sizes (partial η²=0.004‐0.065), with CIs indicating considerable uncertainty around the estimated treatment effects. In contrast, subjective sleep outcomes showed larger adjusted effects. The intervention was associated with a shorter self-reported sleep latency (β=−19.78 min, 95% CI −33.83 to −5.74), corresponding to a large effect size (partial η²=0.194). Sleep duration (β=36.32 min, 95% CI −5.01 to 77.64; partial η²=0.086), sleep efficiency (β=8.75%, 95% CI −0.97 to 18.47; partial η²=0.090), and the global PSQI score (β=−1.20, 95% CI −3.18 to 0.78; partial η²=0.061) also favored the intervention group, although the CIs were compatible with a range of plausible effects. Unadjusted bivariate analyses are provided in Multimedia Appendix 1.

Objective sleep monitoring indicated that the predominant baseline chronotype in both groups was intermediate (ie, neither clearly morning nor evening type). Following the intervention, a shift toward a more evening-oriented chronotype was observed in both groups (Table 4).

Satisfaction with the intervention received and with the participation of the expert (Table 5) was mostly “very good” in all the items evaluated. Satisfaction with the intervention was very good for 63% of the survey and good for 35%.

**Table 3.** Adjusted treatment effects of the group-based cognitive behavioral therapy for insomnia (CBT-I) intervention on circadian rhythm, objective sleep, and subjective sleep outcomes^a^.

Outcome	Adjusted treatment effect (IG^b^ vs CG^c^), β (95% CI)	*P* value	Partial η²
Circadian rhythm			
TAPL^d^ V5	−0.004 (−0.030 to 0.021)	.73	0.004
Interdaily stability (IS)	−0.051 (−0.130 to 0.029)	.25	0.047
Relative amplitude (RAN)	−0.010 (−0.054 to 0.034)	.66	0.006
Environmental synchronization	0.042 (−0.032 to 0.116)	.26	0.037
Circadian health score (CHS)	0.009 (−0.036 to 0.053)	.69	0.005
Objective sleep outcomes			
Temperature 2 h before bedtime	−0.329 (−0.915 to 0.254)	.26	0.037
Temperature during sleep	0.217 (−0.234 to 0.668)	.34	0.027
White light exposure 2 h before bedtime	−0.680 (−3.184 to 1.824)	.59	0.009
Blue light exposure 2 h before bedtime	−0.035 (−0.162 to 0.093)	.59	0.009
Sleep latency	5.63 (−1.80 to 13.06)	.69	0.065
Total sleep time	−7.56 (−47.17 to 32.04)	.70	0.004
Sleep activity time	−0.208 (−0.125 to 0.540)	.21	0.045
Number of awakenings	−0.157 (−0.521 to 0.207)	.39	0.022
Wake after sleep onset	5.14 (−7.81 to 18.10)	.43	0.019
Sleep efficiency	−1.93 (−4.64 to 0.79)	.16	0.058
Results of PSQI^e^			
Sleep latency (min)	−19.78 (−33.83 to −5.74)	.007	0.194
Time in bed	−0.45 (−39.56 to 38.65)	.98	0.001
Sleep time	36.32 (−5.01 to 77.64)	.08	0.086
Sleep efficiency	8.75 (−0.97 to 18.47)	.08	0.090
Global PSQI score	−1.20 (−3.18 to 0.78)	.22	0.061

a Adjusted treatment effects were estimated using analysis of covariance models with the postintervention value as the dependent variable, treatment group as the fixed factor, and the corresponding baseline value, smoking status, school schedule, and coffee consumption as covariates. Partial η² was interpreted as <0.01 (trivial), 0.01 to <0.06 (small), 0.06 to <0.14 (moderate), and ≥0.14 (large).

b IG: intervention group.

c CG: control group.

d TAPL: temperature, acceleration, position, and exposure to light.

e PSQI: Pittsburgh Sleep Quality Index.

**Table 4.** Changes in chronotype distribution before and after a group-based cognitive behavioral therapy for insomnia (CBT-I) intervention among first-year nursing students participating in a pilot randomized controlled trial^a^.

Chronotype	Preintervention		Postintervention	
	IG^b^ (n=20), n (%)	CG^c^ (n=20), n (%)	IG (n=20), n (%)	CG (n=20), n (%)
Extreme matutine	—^d^	—	—	—
Matutine	1 (5)	0 (0)	1 (5)	2 (10)
Undefined	12 (60)	12 (60)	8 (40)	8 (40)
Vespertine	4 (20)	5 (25)	8 (40)	4 (20)
Extreme vespertine	3 (15)	3 (15)	3 (15)	3 (15)

a Pre-post *P *values are IG=.30 and CG=.47.

b IG: intervention group.

c CG: control group.

d Not applicable.

**Table 5.** Results of satisfaction with the intervention^a^.

Variables	Very good (n=20), n (%)	Good (n=20), n (%)	Regular (n=20), n (%)	Bad (n=20), n (%)	Very bad (n=20), n (%)
General assessment^b^					
Dissemination before commencing intervention	9 (45)	11 (55)	0 (0)	0 (0)	0 (0)
Briefing before commencing intervention	7 (35)	13 (65)	0 (0)	0 (0)	0 (0)
Explanation of study goals	16 (80)	4 (20)	0 (0)	0 (0)	0 (0)
Communication between participants and expert	15 (75)	5 (25)	0 (0)	0 (0)	0 (0)
Audiovisual media and materials used	14 (70)	6 (30)	0 (0)	0 (0)	0 (0)
Assessment of intervention expert^b^					
Clarity of presentation	18 (90)	2 (10)	0 (0)	0 (0)	0 (0)
Expert’s knowledge on intervention content	15 (75)	5 (25)	0 (0)	0 (0)	0 (0)
Participant’s motivation level	10 (50)	10 (50)	0 (0)	0 (0)	0 (0)
Adequate number of study activities	11 (55)	8 (40)	1 (5)	0 (0)	0 (0)
Counseling and support during the intervention	12 (60)	8 (40)	0 (0)	0 (0)	0 (0)
Technological resources and applications used	9 (45)	11 (55)	0 (0)	0 (0)	0 (0)
Overall satisfaction with the intervention	13 (65)	7 (35)	0 (0)	0 (0)	0 (0)

a Effect size *r*: 0-0.3 for low, >0.3-0.5 for moderate, and >0.5 for big.

b Participants responded to multiple satisfaction items; therefore, the frequencies within each response category do not add up to the total sample (n=20). For each individual item, responses add up to 20 participants.

## Discussion

### Principal Findings

The present pilot randomized controlled trial explored the preliminary effects of a brief group-based CBT-I–informed intervention delivered as a sleep health promotion program for first-year nursing students. Overall, the intervention showed more favorable effects on subjective sleep outcomes, particularly self-reported sleep latency, whereas objective circadian rhythm and sleep parameters assessed through ACM showed small and imprecise treatment effects. Participants also reported high satisfaction with the intervention, supporting the feasibility and acceptability of implementing this type of program within a university setting.

The pattern of findings observed in this study may be explained by the characteristics of both the intervention and the study population. Unlike most CBT-I trials, which recruit participants with clinically significant insomnia symptoms, this intervention was delivered as a universal health promotion strategy to first-year nursing students regardless of their baseline sleep status [10,12,13]. Consequently, participants may have had relatively limited objective circadian impairment at baseline, reducing the opportunity to detect measurable physiological improvements over the short intervention period. At the same time, increasing participants’ awareness of sleep-related behaviors and promoting healthier sleep habits may have influenced their perception of sleep before producing detectable changes in objective circadian regulation [37].

The characteristics of the intervention itself should also be considered when interpreting these findings. Standard CBT-I programs are typically delivered over multiple sessions and incorporate individualized behavioral components, including stimulus control, sleep restriction, cognitive therapy, and the systematic review of sleep diaries, which are considered core mechanisms of treatment effectiveness [38,39]. By contrast, our intervention consisted of 2 face-to-face group sessions designed as a pragmatic university-based health promotion program rather than as an intensive therapeutic intervention. This lower treatment intensity may have been sufficient to increase participants’ awareness of healthy sleep practices and influence their perception of sleep while being insufficient to induce sustained behavioral changes that could be reflected in objectively measured circadian outcomes during the relatively short follow-up period [40,41]. Furthermore, participant engagement with the behavioral recommendations outside the classroom was not formally assessed. Consequently, it remains unclear to what extent the absence of objective effects reflects the limited intensity of the intervention, suboptimal adherence to the recommended behavioral changes, or a combination of these factors, all of which are recognized determinants of CBT-I effectiveness [39,42].

The discrepancy between subjective and objective outcomes deserves particular attention. Participants in the intervention group reported improvements in sleep latency, with trends toward better perceived sleep duration and sleep efficiency, whereas objective monitoring did not demonstrate corresponding improvements. Similar discrepancies between subjective and objective sleep measures have been described previously in young adults and may reflect the fact that these instruments capture different aspects of sleep [37]. The discrepancy between subjective and objective outcomes should also be interpreted in light of the study design. Because participants were aware of their group allocation and the control group received no active intervention, subjective outcomes were more susceptible to expectancy effects, increased awareness of sleep behaviors, facilitator attention, and group interaction. Consequently, the observed improvements in subjective sleep outcomes may partly reflect nonspecific effects associated with participation in the intervention, in addition to the specific therapeutic components of CBT-I [43]. In contrast, objectively measured circadian outcomes are less directly influenced by participants’ expectations and therefore provide a complementary perspective on intervention effects.

Our findings are broadly consistent with previous studies evaluating CBT-I and behavioral sleep interventions in university students. Randomized trials and systematic reviews have generally reported greater improvements in subjective sleep outcomes than in objective sleep measures, particularly when interventions are implemented in nonclinical student populations [13,40,44]. However, direct comparisons should be interpreted cautiously because studies differ substantially in intervention intensity, treatment duration, participant characteristics, and outcome assessment. Moreover, most previous studies have relied primarily on self-reported questionnaires or conventional actigraphy [13]. By incorporating multidimensional ACM alongside validated subjective measures, the present study provides a more comprehensive evaluation of behavioral intervention effects under real-world conditions and highlights the value of assessing both perceived and physiological dimensions of sleep [45].

Although the objective circadian outcomes did not demonstrate consistent intervention effects, these findings should not be interpreted as evidence that behavioral sleep interventions are ineffective in university populations. Rather, they suggest that a brief group-based health promotion program may be insufficient to modify physiological circadian regulation over a short period in generally healthy young adults. Future trials should evaluate whether greater intervention intensity, individualized behavioral support, reinforcement sessions, longer follow-up, and systematic assessment of participant adherence improve both subjective and objective sleep outcomes [44]. Such research may also help clarify the temporal relationship between changes in perceived sleep and objectively measured circadian outcomes.

One of the main strengths of this study is the combined assessment of subjective sleep quality and multidimensional objective circadian monitoring using a validated ambulatory wearable device under free-living conditions. This approach enabled continuous assessment of sleep and circadian function beyond self-reported outcomes—an aspect that has been infrequently incorporated into behavioral sleep intervention studies in university populations. In addition, the randomized controlled design and the high levels of participant satisfaction support the feasibility and acceptability of implementing sleep health promotion programs among first-year nursing students.

Overall, this pilot trial suggests that a brief, group-based, CBT-I–informed intervention is feasible, well-accepted, and may improve selected subjective aspects of sleep among first-year nursing students. However, measurable changes in objective circadian outcomes were not observed under the conditions evaluated. These findings support the need for adequately powered randomized controlled trials incorporating active control groups, longer follow-up periods, adherence assessment, and more intensive or individualized behavioral interventions to better establish the effectiveness of sleep health promotion programs in university populations.

### Limitations

One of the main limitations of this study was the lack of blinding for participants and facilitators, which is common in behavioral interventions and may introduce bias. Additionally, the absence of an active control group may affect internal validity and potentially lead to an overestimation of the intervention effects. Although the use of a usual-care control enhances ecological validity in a university context, it does not account for nonspecific effects inherent to group-based interventions, such as therapist attention, peer interaction, and participant expectations. Furthermore, the face-to-face group format itself may have contributed to the observed outcomes through contextual or placebo effects, which cannot be ruled out.

The inclusion of an active control condition (eg, group-based education sessions without CBT-I components) would have allowed for more rigorous control over these nonspecific effects. Therefore, the findings should be interpreted with caution. In addition, the group-based format of the intervention did not allow for individualized tailoring based on the specific sleep characteristics identified during preintervention assessment. Although key aspects of sleep behavior were addressed, more personalized approaches may yield stronger effects.

Furthermore, although mediation analysis was prespecified in the study protocol, it was not performed due to the limited sample size and the absence of multiple follow-up assessments required to establish temporal relationships between potential mediators and outcomes. As a result, the mechanisms through which the intervention may have influenced sleep- and circadian-related outcomes could not be explored. Future, adequately powered trials that incorporate repeated measurements over time should investigate potential mediating pathways underlying intervention effects.

In addition, the study may be subject to selection bias. Participants were recruited from a single cohort of first-year nursing students at one university, and participation was voluntary. Consequently, students with greater interest in sleep-related issues or health-promoting behaviors may have been more likely to participate. This may limit the representativeness of the sample and reduce the generalizability of the findings to other student populations, academic disciplines, or educational settings.

Although participants were randomly allocated, some baseline differences were observed between groups in variables such as smoking status, school schedules, and caffeine consumption. While additional adjusted analyses were performed and yielded results consistent with the primary findings, residual confounding cannot be completely excluded given the small sample size characteristic of this pilot study.

Future research should aim to incorporate active control conditions, larger sample sizes, and more individualized interventions. Further studies are also needed to determine the clinical relevance of changes observed in circadian parameters and to validate multidisciplinary approaches based on individual sleep and circadian profiles. Finally, this pilot trial was not intended to address implementation at the institutional level. Future implementation studies are needed to evaluate scalability, resource requirements, and cost-effectiveness before broader adoption can be considered.

### Conclusions

This pilot randomized controlled trial provides preliminary evidence that a brief group-based CBT-I intervention may improve selected subjective sleep outcomes among first-year nursing students. The largest treatment effect was observed for self-reported sleep latency, whereas objective circadian rhythm and sleep outcomes generally showed small treatment effects with wide CIs, reflecting considerable uncertainty around the estimated effects. Participants reported high levels of satisfaction with the intervention, supporting the feasibility and acceptability of this approach within a university setting. Given the exploratory nature and limited sample size of this pilot study, adequately powered randomized controlled trials with longer follow-up are needed to confirm these findings and to better define the magnitude and durability of the intervention effects.

## Supplementary material

10.2196/85021 Multimedia Appendix 1 Unadjusted bivariate analyses of circadian rhythm parameters, sleep, and subjective sleep quality.

10.2196/85021 Checklist 1 CONSORT 2010 checklist.

## Abbreviations

ACM: ambulatory circadian monitoring
CBT: cognitive behavioral therapy
CBT-I: cognitive behavioral therapy for insomnia
CHS: circadian health score
CONSORT: Consolidated Standards of Reporting Trials
ES: environmental synchronization
IPAQ: International Physical Activity Questionnaire
IS: interdaily stability
PSQI: Pittsburgh Sleep Quality Index
RAN: relative amplitude normalized
TAPL: temperature, acceleration, position, and exposure to light

## References

[1] Wittmann M, Dinich J, Merrow M, Roenneberg T (2006). Social jetlag: misalignment of biological and social time. Chronobiol Int.

[2] Ohayon M, Wickwire EM, Hirshkowitz M (2017). National Sleep Foundation’s sleep quality recommendations: first report. Sleep Health.

[3] Kessler RC, Berglund PA, Coulouvrat C (2012). Insomnia, comorbidity, and risk of injury among insured Americans: results from the America Insomnia Survey. Sleep.

[4] Nelson KL, Davis JE, Corbett CF (2022). Sleep quality: an evolutionary concept analysis. Nurs Forum.

[5] Mellinger GD, Balter MB, Uhlenhuth EH (1985). Insomnia and its treatment. Prevalence and correlates. Arch Gen Psychiatry.

[6] Howell AJ, Jahrig JC, Powell RA (2004). Sleep quality, sleep propensity and academic performance. Percept Mot Skills.

[7] Silva M, Chaves C, Duarte J, Amaral O, Ferreira M (2016). Sleep quality determinants among nursing students. Procedia Soc Behav Sci.

[8] Chan CKL, So WKW, Fong DYT (2009). Hong Kong baccalaureate nursing students’ stress and their coping strategies in clinical practice. J Prof Nurs.

[9] Krystal AD, Prather AA, Ashbrook LH (2019). The assessment and management of insomnia: an update. World Psychiatry.

[10] Koffel EA, Koffel JB, Gehrman PR (2015). A meta-analysis of group cognitive behavioral therapy for insomnia. Sleep Med Rev.

[11] Irish LA, Kline CE, Gunn HE, Buysse DJ, Hall MH (2015). The role of sleep hygiene in promoting public health: a review of empirical evidence. Sleep Med Rev.

[12] Murawski B, Wade L, Plotnikoff RC, Lubans DR, Duncan MJ (2018). A systematic review and meta-analysis of cognitive and behavioral interventions to improve sleep health in adults without sleep disorders. Sleep Med Rev.

[13] Friedrich A, Schlarb AA (2018). Let’s talk about sleep: a systematic review of psychological interventions to improve sleep in college students. J Sleep Res.

[14] Blazquez A, Martinez-Nicolas A, Salazar FJ, Rol MA, Madrid JA (2012). Wrist skin temperature, motor activity, and body position as determinants of the circadian pattern of blood pressure. Chronobiol Int.

[15] Yilmaz D, Tanrikulu F, Dikmen Y (2017). Research on sleep quality and the factors affecting the sleep quality of the nursing students. Curr Health Sci J.

[16] Edinger JD, Means MK (2005). Cognitive-behavioral therapy for primary insomnia. Clin Psychol Rev.

[17] Buysse DJ, Reynolds 3rd CF, Monk TH, Berman SR, Kupfer DJ (1989). The Pittsburgh Sleep Quality Index: a new instrument for psychiatric practice and research. Psychiatry Res.

[18] Ortiz‐Tudela E, Martinez‐Nicolas A, Albares J (2014). Ambulatory circadian monitoring (ACM) based on thermometry, motor activity and body position (TAP): a comparison with polysomnography. Physiol Behav.

[19] Mullington JM, Abbott SM, Carroll JE (2016). Developing biomarker arrays predicting sleep and circadian-coupled risks to health. Sleep.

[20] Ortiz-Tudela E, Martinez-Nicolas A, Campos M, Rol MÁ, Madrid JA (2010). A new integrated variable based on thermometry, actimetry and body position (TAP) to evaluate circadian system status in humans. PLoS Comput Biol.

[21] Martinez-Nicolas A, Martinez-Madrid MJ, Almaida-Pagan PF, Bonmati-Carrion MA, Madrid JA, Rol MA (2019). Assessing chronotypes by ambulatory circadian monitoring. Front Physiol.

[22] Ruiz-Zaldibar C, Gal-Iglesias B, Azpeleta-Noriega C, Ruiz-López M, Pérez-Manchón D (2022). The effect of a sleep intervention on sleep quality in nursing students: study protocol for a randomized controlled trial. Int J Environ Res Public Health.

[23] Morin CM, Benca R (2012). Chronic insomnia. Lancet.

[24] Whitehead AL, Julious SA, Cooper CL, Campbell MJ (2016). Estimating the sample size for a pilot randomised trial to minimise the overall trial sample size for the external pilot and main trial for a continuous outcome variable. Stat Methods Med Res.

[25] Julious SA (2005). Sample size of 12 per group rule of thumb for a pilot study. Pharm Stat.

[26] Madrid-Navarro CJ, Puertas Cuesta FJ, Escamilla-Sevilla F (2019). Validation of a device for the ambulatory monitoring of sleep patterns: a pilot study on Parkinson’s disease. Front Neurol.

[27] Madrid-Navarro CJ, Escamilla-Sevilla F, Mínguez-Castellanos A (2018). Multidimensional circadian monitoring by wearable biosensors in Parkinson’s disease. Front Neurol.

[28] Sarabia JA, Rol MA, Mendiola P, Madrid JA (2008). Circadian rhythm of wrist temperature in normal-living subjects. A candidate of new index of the circadian system. Physiol Behav.

[29] Raymann RJEM, Swaab DF, Van Someren EJW (2005). Cutaneous warming promotes sleep onset. Am J Physiol Regul Integr Comp Physiol.

[30] Carskadon MA, Dement WC, Kryger MH, Roth T, Dement WC (2011). Principles and Practice of Sleep Medicine.

[31] Juda M, Vetter C, Roenneberg T (2013). The Munich ChronoType Questionnaire for Shift-Workers (MCTQShift). J Biol Rhythms.

[32] de la Vega R, Tomé-Pires C, Solé E (2015). The Pittsburgh Sleep Quality Index: validity and factor structure in young people. Psychol Assess.

[33] Craig CL, Marshall AL, Sjöström M (2003). International Physical Activity Questionnaire: 12-country reliability and validity. Med Sci Sports Exerc.

[34] Azpeleta C, Santos P, Sobrado A, Lesmes M, Gal B (2021). Forcing a change: a learn-by-doing workshop on circadian rhythms to understand the complexities of human physiology. Adv Physiol Educ.

[35] Cohen J (1988). Statistical Power Analysis for the Behavioral Sciences.

[36] Rosenthal R, Rubin DB (1991). Further issues in effect size estimation for one-sample multiple-choice-type data. Psychol Bull.

[37] Albinni B, Baker FC, Javitz H (2023). Morning perception of sleep, stress, and mood, and its relationship with overnight physiological sleep: findings from the National Consortium on Alcohol and Neurodevelopment in Adolescence (NCANDA) study. J Sleep Res.

[38] Edinger JD, Arnedt JT, Bertisch SM (2021). Behavioral and psychological treatments for chronic insomnia disorder in adults: an American Academy of Sleep Medicine clinical practice guideline. J Clin Sleep Med.

[39] Walker J, Muench A, Perlis ML, Vargas I (2022). Cognitive behavioral therapy for insomnia (CBT-I): a primer. Klin Spec Psihol.

[40] Tadros M, Newby JM, Li S, Werner-Seidler A (2025). A systematic review and meta-analysis of psychological treatments to improve sleep quality in university students. PLoS ONE.

[41] Chandler L, Patel C, Lovecka L (2022). Improving university students’ mental health using multi-component and single-component sleep interventions: a systematic review and meta-analysis. Sleep Med.

[42] Fretz KM (2023). Recommendations to bolster adherence in cognitive behavioral therapy for insomnia: a self-efficacy approach. Transl Behav Med.

[43] Yeung V, Sharpe L, Geers A, Colagiuri B (2020). Choice, expectations, and the placebo effect for sleep difficulty. Ann Behav Med.

[44] Lu YA, Lin HC, Tsai PS (2025). Effects of digital sleep interventions on sleep among college students and young adults: systematic review and meta-analysis. J Med Internet Res.

[45] Bonmati-Carrion MA, Middleton B, Revell V, Skene DJ, Rol MA, Madrid JA (2014). Circadian phase assessment by ambulatory monitoring in humans: correlation with dim light melatonin onset. Chronobiol Int.

