# Supplementary material for: Effects of a Group-Based Cognitive Behavioral Therapy for Sleep in Nursing Students: Pilot Randomized Controlled Trial
Source: JMIR Hum Factors. 2026 Sep 16;13:e85021. doi: 10.2196/85021 (PMC13581143; doi:10.2196/85021)
Supplement: Supplementary file 2 [file humanfactors-v13-e85021-s001.docx]

Supplementary Table S1. Unadjusted bivariate analyses of circadian rhythm parameters.

|  | **Pre-Intervention** | | **Post-Intervention** | | **Pre-post** | **Differences**  **(median ± IQR [range])** | |  |
| --- | --- | --- | --- | --- | --- | --- | --- | --- |
|  | **IG (n = 20)** | **CG (n=20)** | **IG (n = 20)** | **CG (n=20)** | **p-value** | **IG (n = 20)** | **CG (n=20)** | |
| **TAPL V5^a^**  **(median ± IQR [range])** | 0.06 ± 0.07  (0.02 – 0.19) | 0.04 ± 0.02  (0.02 – 0.06) | 0.05 ± 0.02  (0.03 – 0.10) | 0.05 ± 0.06  (0.03 – 0.15) | **IG**: .064 | -0.01 ± 0.05  (-0.15 to 0.07) | 0.01 ± 0.05  (-0.03 to 0.12) | |
|  |  |  |  |  | **CG**: .173 |  |  |  |
| **p -value / effectsize** |  | |  | |  | **p=.011 / r=0.4**  **(0.11 to 0.634)** | | |
| **IS TAP^b^**  **(median ± IQR [range])** | 0.41 ± 0.1  (0.2 – 0.6) | 0.52 ± 0.19  (0.32 – 0.69) | 0.44 ± 0.17  (0.31 – 0.58) | 0.49 ± 0.14  (0.21 – 0.65) | **IG**: .131 | 0.03 ± 0.17  (-0.22 to 0.31) | -0.03 ± 0.26  (-0.48 to 0.33) | |
|  |  |  |  |  | **CG**: .161 |  |  |  |
| **p -value / effectsize** |  | |  | |  | **p=.042 / r=0.32**  **(0.01 to 0.575)** | | |
| **RAN TAP^c^**  **(median ± IQR [range])** | 0.41 ± 0.09  (0.24 – 0.48) | 0.48 ± 0.08  (0.15 – 0.56) | 0.43 ± 0.08  (0.33 – 0.51) | 0.43 ± 0.09  (0.25 – 0.51) | **IG:** .209 | 0.01 ± 0.09  (-0.12 to 0.19) | -0.03 ± 0.15  (-0.31 to 0.36) | |
|  |  |  |  |  | **CG**: .150 |  |  |  |
| **p -value / effectsize** |  | |  | |  | p=.101 / r=0.26  (-0.06 to 0.528) | | |
| **ES TAP^d^**  **(median ± IQR [range])** | 0.77 ± 0.15  (0.5 – 1) | 0.76 ± 0.15  (0.3 – 0.9) | 0.74 ± 0.13  (0.52 – 0.94) | 0.76 ± 0.19  (0.47 – 0.96) | **IG:** .779 | 0 ± 0.21  (-0.29 to 0.22) | 0.01 ± 0.21  (-0.24 to 0.21) | |
|  |  |  |  |  | **CG**: .888 |  |  |  |
| **p -value / effectsize** |  | |  | |  | p=.695 / r=0.06  (-0.25 to 0.367) | | |
| **CHS TAP^e^**  **(median ± IQR [range])** | 0.63 ± 0.08  (0.51 - 0.73) | 0.68 ± 0.06  (0.54 – 0.74) | 0.66 ± 0.07  (0.54 – 0.72) | 0.66 ± 0.08  (0.49 – 0.73) | **IG:** .254 | 0.02 ± 0.11  (-0.12 to 0.17) | - 0.03 ± 0.1  (-0.22 to 0.18) | |
|  |  |  |  |  | **CG**: .079 |  |  |  |
| **p -value / effectsize** |  | |  | |  | **p=.025 / r=0.35**  **(0.047 to 0.599)** | | |

^a^ TAPL V5: Wrist temperature, activity, position, light exposure (5^th^:0,03 – 95^th^:0,07). ^b^ IS TAP: Interdaily Stability (5^th^:0,27 – 95^th^:0,60). ^c^RANorm TAP: Relative Amplitude Normalized (5^th^:0,2 – 95^th^:0,6). ^d^ES TAP: Environmental Synchronization (5^th^:0,65 - ). ^e^ CHS TAP: Circadian Health System (5^th^:0,60 - ). Effect size r: 0–0.3 low, > 0.3–0.5 moderate, and > 0.5 big effect size. IC: Intervention Group; CG: Control Group

Supplementary Table S2. Unadjusted bivariate analyses of sleep.

|  | Pre-Intervention | | | Post-Intervention | | | Pre-post | Differences  (mean ± SD [range]) | | |
| --- | --- | --- | --- | --- | --- | --- | --- | --- | --- | --- |
|  | **IG (n = 20)** | **CG (n=20)** | | **IG (n = 20)** | | **CG (n=20)** | **p-value** | **IGI (n = 20)** | **CG (n=20)** | |
| Temperature 2h before  (mean ± SD [range]) | 31.2 ± 1.4  (28.4 – 33.5) | 31.1 ± 1.4  (28.2 – 33.5) | | 31.2 ± 1.3  (28.2 – 33.1) | | 31.5 ± 0.9  (29.7 – 32.9) | **IG:** .810 | -0.5 ± 0.9  (-2 to 1.9) | 0.4 ± 1  (-1.9 to 2.6) | |
|  |  |  |  |  |  |  | **CG**: .107 |  |  |  |
| p -value /effect size |  | | |  | | |  | p=.165 / **d=1.4**  (-2.93 to 1.60) | | |
| Temperature during sleep  (mean ± SD [range]) | 33.6 ± 0.8  (32.3 – 35.7) | 33.8 ± 0.7  (32.2 – 35.3) | | 33.6 ± 0.9  (31.8 – 36.1) | | 33.7 ± 0.8  (31.9 – 35.5) | **IG:** .953 | 0.01 ± 0.5  (-0.9 to 1.1) | -0.08 ± 0.7  (-1.9 to 0.9) | |
|  |  |  |  |  |  |  | **CG**: .583 |  |  |  |
| p -value /effect size |  | | |  | | |  | p=.636 / **d=1.48**  (-0.473 to 1.769) | | |
| White light 2h before  (mean ± SD [range]) | 9.9 ± 3.2  (4.7 – 16.5) | 10.2 ± 2.8  (5 – 14.2) | | 10.3 ± 3.7  (2.9 – 18.3) | | 11.3 ± 4.3  (0 – 17) | **IG:** .600 | 0.4 ± 3.3  (-5.2 to 9.9) | 1.1 ± 3.8  (-9.9 to 6.5) | |
|  |  |  |  |  |  |  | **CG**: .210 |  |  |  |
| p -value /effect size |  | | |  | | |  | p=.539 / d=0.19  (--0.818 to 0.425) | | |
| Blue light 2h before  (mean ± SD [range]) | 0.43 ± 0.22  (0.12 – 0.88) | 0.38 ± 0.20  (0.02 – 0.71) | | 0.37 ± 0.20  (0.1 – 0.77) | | 0.40 ± 0.2  (0.13 – 0.85) | **IG:** .209 | -0.06 ± 0.2  (-0.04 to 0.4) | 0.02 ± 0.19  (-0.02 to 0.5) | |
|  |  |  |  |  |  |  | **CG**: .619 |  |  |  |
| p -value /effect size |  | | |  | | |  | p=.199 / **d=0.59**  (-0.216 to 1.036) | | |
| Sleep latency  (mean ± SD [range]) | 30.8 ± 13.4  (6 - 58) | | 25.1 ± 11.6  (12 – 54) | 27.6 ± 10.3  (14 – 52) | 22.1 ± 8.5  (9 – 37) | | **IG:** .456 | -3.15 ± 18.5  (-44 to 24) | | -3 ± 12.6  (-34 to 23) |
|  |  |  |  |  |  |  | **CG**: .310 |  |  |  |
| p -value /effect size |  | | |  | | |  | p=.968 / **d=1.97**  (-0.629 to 2.11) | | |
| Total sleep time  (mean ± SD [range]) | 420.3 ± 39.1  (341 – 510) | | 425.8 ± 48.3  (320 – 538) | 408.3 ± 58.9  (260 – 495) | 425.3 ± 46.6  (307 – 502) | | **IG:** .430 | -12 ± 66.6  (-153 to 109) | | -0.5 ± 63  (-167 to 71) |
|  |  |  |  |  |  |  | **CG**: .972 |  |  |  |
| p -value /effect size |  | | |  | | |  | p=.578 / d=0.01 | | |
| Sleep activity time  (mean ± SD [range]) | 1.2 ± 0.3  (0.7 – 1.96) | | 1.4 ± 06  (0.6 – 2.56) | 1.4 ± 0.4  (0.7 - 2.13) | 1.4 ± 0.6  (0.75 – 2.63) | | **IG: .021** | 0.22 ± 0.39  (-0.42 to 1) | | 0.04 ± 0.64  (1-1.4 to 1.1) |
|  |  |  |  |  |  |  | **CG**: .797 |  |  |  |
| p -value /effect size |  | | |  | | |  | p=.287 / **d=0.34**  (-0.284 to 0.964) | | |
| Awakenings  (mean ± SD [range]) | 3 ± 0.6  (1.8 – 4.3) | | 2.9 ± 0.7  (1.7 – 4.5) | 3.2 ± 0.8  (1.9 – 4.9) | 3 ± 0.7  (2 – 4.4) | | **IG:** .347 | 0.11 ± 0.52  (-0.9 to 1.6) | | 0.16 ± 0.47  (-0.5 to 1.1) |
|  |  |  |  |  |  |  | **CG**: .133 |  |  |  |
| p -value /effect size |  | | |  | | |  | p=.745 / d=0.1  (-0.721 to 0.519) | | |
| WASO  (mean ± SD [range]) | 50.35 ± 15.3  (20 - 90) | | 49.75 ± 17.5  (21 - 82) | 55.05 ± 17.1  (25 - 90) | 54.1 ± 22.5  (26 - 115) | | **IG:** .177 | 4.7 ± 15  (-24 to 33) | | 4.35 ± 23.4  (-39 to 40) |
|  |  |  |  |  |  |  | **CG**: .416 |  |  |  |
| p -value /effect size | .909 | | | .881 | | |  | P=.955 / d=0.02  (-0.602 to 0.638) | | |
| Sleep Efficiency  (mean ± SD [range]) | 81.5 ± 4.4  (71.4 – 90.4) | | 83.7 ± 3.9  (78.5 – 91.5) | 80.8 ± 3.4  (74 – 85.5) | 82.9 ± 3.6  (76.5 – 89.3) | | **IG:** .556 | -0.64 ± 4.8  (-9.2 to 7.1) | | -0.86 ± 4.8  (-15 to 7) |
|  |  |  |  |  |  |  | **CG**: .433 |  |  |  |
| p -value /effect size |  | | |  | | |  | p=.888 / **d=0.46**  (-0.574 to 0.666) | | |

Supplementary Table S3. Unadjusted bivariate analyses of subjective sleep quality assessed using the Pittsburgh Sleep Quality Index (PSQI)

|  | | Pre-Intervention | | Post-Intervention | | Pre-post | Differences  (median ± IQR [range]) | |
| --- | --- | --- | --- | --- | --- | --- | --- | --- |
|  | | **IG (n = 20)** | **CG (n=20)** | **IG (n = 20)** | **CG (n=20)** | **p-value** | **IG (n = 20)** | **CG (n=20)** |
| Sleep latency (min)  (median ± IQR [range]) | | 25 ± 30  (5 – 60) | 30 ± 45  (1 – 90) | 15 ± 26  (1 – 60) | 37.5 ± 44  (5 – 120) | **IG:** .420 | 0 ± 20  (-59 to 30) | 0 ± 34.3  (-45 to 60) |
|  |  |  |  |  |  | **CG**: .248 |  |  |
| p -value / effect size | |  | |  | |  | p=.240 / r=0.19  (-0.130 to 0.472) | |
| Latency categorized | ≤ 15 min | 7 (35%) | 7 (35%) | 10 (50%) | 5 (25%) | **IG:** .532 |  | |
|  | 16 – 30 min | 7 (35%) | 4 (20%) | 6 (30%) | 5 (25%) |  |  |  |
|  | 31 – 60 min | 6 (30%) | 5 (25%) | 4 (20%) | 6 (30%) | **CG**: .675 |  |  |
|  | > 60 min | 0 | 4 (20%) | 0 | 4 (20%) |  |  |  |
| Time in bed (min)  (median ± IQR [range]) | | 415.5 ± 140  (280 – 600) | 510 ± 90  (390 – 570) | 420 ± 93.8  (310 – 525) | 480 ± 142.5  (360 – 690) | **IG:** .913 | 0 ± 86.3  (-150 to 105) | 0 ± 101.3  (-150 to 270) |
|  |  |  |  |  |  | **CG**: .534 |  |  |
| p -value / effect size | |  | |  | |  | p=.234 / r=0.03  (-0.307) to 0.312) | |
| Sleep time (min)  (median ± IQR [range]) | | 360 ± 120  (180 – 480) | 420 ± 105  (240 – 480) | 390 ± 60  (300 – 480) | 420 ± 165  (240 – 540) | **IG: .020** | 0 ± 60  (-60 to 120) | 0 ± 120  (-180 to 120) |
|  |  |  |  |  |  | **CG**: .886 |  |  |
| p -value / effect size | |  | |  | |  | p=.212 /r=0,20  (-0.119 to 0.481) | |
| Categorized  sleep time | > 7 horas | 2 (10%) | 5 (25%) | 3 (15%) | 6 (30%) | **IG:** .675 |  | |
|  | 6 – 7 horas | 9 (45%) | 8 (40%) | 11 (55%) | 6 (30%) |  |  |  |
|  | 5 – 6 horas | 7 (35%) | 7 (35%) | 6 (30%) | 4 (20%) | **CG**: .370 |  |  |
|  | < 5 horas | 2 (10%) | 0 | 0 | 4 (20%) |  |  |  |
| Sleep efficiency (%)  (median ± IQR [range]) | | 88.3 ± 27.3  (50 – 114.3) | 82.4 ± 16.3  (44.4 – 106.7) | 88.9 ± 10.6  (80 – 103.7) | 86.6 ± 30.8  (50 – 116.7) | **IG:** .099 | 4.3 ± 24  (-14.3 to 38.2) | -2.6 ± 25.8  (-32.4 to 33.9) |
|  |  |  |  |  |  | **CG**: .778 |  |  |
| p -value / effect size | |  | |  | |  | p=.372 / r=0.14  (-0.177 to 0.434) | |
| Sleep disturbance | None | 4 (20%) | 0 | 3 (15%) | 0 | **IG:** .717 |  | |
|  | <1 per week | 15 (75%) | 13 (65%) | 15 (75%) | 18 (90%) |  |  | |
|  | 1-2 times/week | 1 (5%) | 7 (35%) | 2 (10%) | 2 (10%) | **CG**: .460 |  | |
|  | ≥ 3 times/week | 0 | 0 | 0 | 0 |  |  | |
| Sleep quality | Quite good | 1 (5%) | 2 (10%) | 1 (5%) | 2 (10%) | **IG:** .532 |  | |
|  | Good | 11 (55%) | 8 (40%) | 11 (55%) | 7 (35%) |  |  | |
|  | Bad | 7 (35%) | 8 (40%) | 7 (35%) | 9 (45%) | **CG:** .910 |  | |
|  | Very bad | 1 (5%) | 2 (10%) | 1 (5%) | 2 (10%) |  |  | |
| Daytime dysfunction | NONE | 1 (5%) | 2 (10%) | 2 (10%) | 1 (5%) | **IG:** .736 |  | |
|  | <1 per week | 7 (35%) | 7 (35%) | 7 (35%) | 8 (40%) |  |  | |
|  | 1-2 times/week | 11 (55%) | 6 (30%) | 9 (45%) | 9 (45%) | **CG**: .225 |  | |
|  | ≥ 3 times/week | 1 (5%) | 5 (25%) | 2 (10%) | 2 (10%) |  |  | |
| Global results  (median ± IQR [range]) | | 8 ± 5  (2 – 14) | 9 ± 5.5  (3 – 17) | 7.5 ± 2.8  (2 – 11) | 8.5 ± 6.8  (4 – 16) | **IG:** .514  **CG**: .654 | -0.5 ± 4  (-6 to 3) | -1 ± 4.3  (-5 to 7) |
|  |  |  |  |  |  |  |  |  |
| p-value / effect size | |  |  |  |  |  | p=.967 / r=0.01  (-0.303 to 0.316) | |
| Good sleep quality* | | 1 (5%) | 2 (10%) | 2 (10%) | 2 (10%) | **IG:** .999 |  | |
| Poor sleep quality** | | 19 (95%) | 18 (90%) | 18 (90%) | 18 (90%) | **CG**: .999 |  | |

Effect size: r: 0–0.3 low, > 0.3–0.5 moderate, and > 0.5 big effect size.

IG: Intervention Group; CG: Control Group
